# Neighborhood Factors and Treatment Outcomes in Pediatric Acute Lymphoblastic Leukemia: A REDIAL Consortium Report

**DOI:** 10.1158/2767-9764.CRC-26-0134

**Published:** 2026-09-04

**Authors:** Rutu A. Rathod, Amy E. Hughes, Pagna Sok, Karen R. Rabin, Sandi L. Pruitt, Philip J. Lupo, Michael E. Scheurer, Jeremy M. Schraw, the REDIAL Consortium

**Affiliations:** 1 Department of Epidemiology, Fay W. Boozman College of Public Health, University of Arkansas for Medical Sciences, Little Rock, Arkansas.; 2 Winthrop P. Rockefeller Cancer Institute, University of Arkansas for Medical Sciences, Little Rock, Arkansas.; 3 Peter O’Donnell School of Public Health, University of Texas Southwestern Medical Center, Dallas, Texas.; 4 Harold C. Simmons Comprehensive Cancer Center, University of Texas Southwestern Medical Center, Dallas, Texas.; 5 Division of Hematology-Oncology, Department of Pediatrics, Baylor College of Medicine, Houston, Texas.; 6 Dan L. Duncan Comprehensive Cancer Center, Baylor College of Medicine, Houston, Texas.; 7 Department of Pediatrics, Benioff Children’s Hospitals, University of California San Francisco, San Francisco, California.; 8 Department of Pediatrics, Emory University School of Medicine, Atlanta, Georgia.; 9 Aflac Cancer and Blood Disorders Center, Children’s Healthcare of Atlanta, Atlanta, Georgia.

## Abstract

Acute lymphoblastic leukemia (ALL) shows persistent outcome disparities among socioeconomically disadvantaged and Hispanic children. Neighborhood socioeconomic status (nSES) and residence in Hispanic enclaves may contribute to these disparities, yet their impact on pediatric ALL is unclear. We examined associations of nSES and Hispanic enclave residence with ALL treatment outcomes. We analyzed children (0–24 years) diagnosed with ALL between 2005 and 2017 and treated at REducing Disparities In Acute Leukemia (REDIAL) Consortium centers in Texas. Census-tract nSES (Yost) and Hispanic enclave indices were computed from US Census and American Community Survey (ACS) data, classifying tracts into low/high nSES and enclave/no enclave residence. Outcomes included end-of-induction (EOI) minimal residual disease (MRD) positivity (MRD ≥ 0.01%), relapse, event-free survival (EFS), and overall survival (OS). Associations were evaluated using multivariable logistic and Cox regression models adjusting for sex, age at diagnosis, race/ethnicity, EOI MRD, cytogenetic features, National Cancer Institute (NCI) risk group at diagnosis, ALL immunophenotype, and treating institutions. Among 1,348 patients, 41% resided in low-nSES tracts and 42% in Hispanic enclaves. Neither enclave residence nor low nSES was associated with EOI MRD positivity; similar null associations were observed for relapse, EFS, and OS. Although unadjusted analyses indicated slightly poorer 5-year EFS for low-nSES areas (81% vs. 85%, *P* = 0.04), this difference attenuated after adjustment. In this large, multicenter, and diverse cohort of children with ALL, neighborhood socioeconomic disadvantage and Hispanic enclave residence were not independently associated with treatment outcomes after accounting for clinical and cytogenetic features. Future studies should examine other social determinants and neighborhood influences on disease characteristics, survivorship, and late effects.

**Significance:**

In this multicenter study, neither neighborhood socioeconomic disadvantage nor residence in a Hispanic/Latino enclave was associated with inferior outcomes among children with ALL after controlling for clinical and cytogenetic disease features. Disparities affecting economically disadvantaged or minoritized populations may be mediated by other factors.

## Introduction

Acute lymphoblastic leukemia (ALL) is the most common pediatric cancer, with an incidence of about 30 cases per million US children and adolescents (1). Despite significant therapeutic advances, socioeconomically disadvantaged, Black, and Hispanic children continue to experience poorer outcomes (2–7). In the United States, race, ethnicity, socioeconomic status, and residential context are interconnected (8). Many Hispanic/Latino (hereafter, “Hispanic”) individuals live in ethnic enclaves, i.e., culturally and ethnically distinct neighborhoods with high concentrations of individuals of same ethnic origin, recent immigrants, linguistically isolated households, and ethnic businesses, often marked by socioeconomic disadvantage (9). There is growing concern that structural and contextual factors, such as neighborhood socioeconomic status (nSES) and Hispanic enclave residence, may contribute to suboptimal health outcomes, yet their influence on treatment outcomes in pediatric ALL remains poorly understood.

Previous studies using population-based registries have examined neighborhood characteristics in relation to pediatric ALL survival, with inconsistent results (10, 11). Despite epidemiologic evidence linking social determinants of health to pediatric cancer outcome disparities, the role of neighborhood-level factors in pediatric ALL outcomes has received limited attention (12, 13). Importantly, registry-based studies are not suitable for studying outcomes like end-of-induction (EOI) minimal residual disease (MRD) positivity, relapse, and treatment-related toxicities. They also often lack important clinical and molecular covariates such as genetic alterations, which may differ between racial and ethnic groups and often strongly influence treatment outcomes (14–20).

Neighborhood socioeconomic conditions and ethnic enclave environments may influence pediatric ALL outcomes through multiple interconnected pathways. Socioeconomically disadvantaged neighborhoods may experience reduced healthcare access, transportation barriers, housing instability, caregiver financial strain, and limited access to supportive resources that could affect treatment adherence, timely management of complications, and continuity of care (21–24). Hispanic enclave residence may similarly influence outcomes through both adverse and protective mechanisms (25). Although enclaves may reflect concentrated disadvantage, linguistic isolation, or barriers to healthcare navigation (26), they may also provide culturally aligned social support, stronger family networks, and community cohesion that facilitate adherence and engagement with care (27). Because these neighborhood environments likely operate through multiple structural and social mechanisms rather than a single pathway, composite neighborhood indices may help capture the cumulative contextual conditions experienced by children undergoing ALL treatment.

Understanding whether nSES and Hispanic enclave residence influence MRD positivity, relapse, overall survival (OS), and event-free survival (EFS) is critical for addressing persistently poorer outcomes for economically disadvantaged and Hispanic children with ALL. To address these gaps, we evaluated associations of nSES and Hispanic enclave residence with (i) MRD positivity at the EOI, (ii) relapse, (iii) OS, and (iv) EFS independent of established clinical and molecular prognostic factors using clinically well-annotated, retrospective data from a large cohort of pediatric patients with ALL.

## Materials and Methods

### Data

The REducing Disparities In Acute Leukemia (REDIAL) Consortium was established to address persistent inferior survival and increased treatment-related toxicities in Hispanic children with leukemia. During data collection for this study, the consortium consisted of six Texas pediatric cancer treatment centers: Texas Children’s Hospital (TCH; Houston); Children’s Health Dallas (Dallas); Cook Children’s (Fort Worth); Vannie E. Cook Jr Children’s Cancer and Hematology Clinic (McAllen); Children’s Hospital of San Antonio (San Antonio); and Texas Tech University Health Sciences Center (El Paso). Data on demographics, clinical and molecular features, residential address at the time of diagnosis, and treatment outcomes were extracted from participants’ electronic health records and entered a central database at Baylor College of Medicine. This study was approved by the Institutional Review Board (IRB) of Baylor College of Medicine and conducted in accordance with recognized ethical guidelines (US Common Rule, 45 CFR 46). The IRB waived the requirement for written informed consent because this retrospective study posed no more than minimal risk and would have been impracticable without the waiver.

We included three pediatric oncology centers in the REDIAL Consortium (TCH, Cook Children’s, and Vannie Cook Clinic) with complete data integration at the time of this analysis. Within these centers, we included patients ≤24 years of age newly diagnosed with ALL from 2005 to 2017. Address data necessary for neighborhood exposure assessment were unavailable or incomplete for some patients and were excluded (*N* = 107; by center: TCH = 41; Cook = 43; Vannie Cook = 23).

### Outcomes

We evaluated the following clinical outcomes: (i) EOI MRD positivity, defined as ≥0.01% residual leukemic blasts detected by flow cytometry in the day 29 bone marrow sample, and when this determination was not possible (e.g., MRD is “negative” or “<0.1%”), it was defined using treatment protocol–specific cutoffs of ≥0.01% or ≥0.1%; (ii) relapse (yes/no); (iii) OS, defined as the time from diagnosis to death or last follow-up (January 15, 2021); and (iv) EFS, defined as the time from diagnosis to ALL relapse, death, or last follow-up.

### Neighborhood measures

We estimated tract-level socioeconomic status and ethnic enclave status using the 2000 decennial census for children diagnosed before 2008, the 2008 to 2012 American Community Survey (ACS) 5-year estimates for children diagnosed between 2008 and 2012, and the 2012 to 2016 ACS 5-year estimates for children diagnosed after 2012. We computed Yost index scores from principal component analysis (PCA) of educational attainment, poverty, income, employment, occupation, home value, and rent (28, 29) to estimate nSES. Values were categorized into state-standardized quintiles, with Q1 to Q2 classified as low nSES and Q3 to Q5 classified as high nSES. We selected the Yost index because it is validated, recommended by Surveillance, Epidemiology, and End Results, and among the most widely used neighborhood socioeconomic measures in cancer epidemiology and has been extensively applied in studies of cancer incidence, treatment, and survival. PCA-based neighborhood indices are designed to summarize multiple correlated socioeconomic characteristics into a multidimensional contextual measure while reducing collinearity across individual census tracts. A recent review of nSES measures in cancer research have noted that PCA-derived indices are commonly used because they capture broader structural and social conditions that may not be adequately represented by any single neighborhood variable (30). We computed the Hispanic ethnic enclave index using methods that have been described (31) and used previously (12, 32) in cancer research. Briefly, we used PCA of four census measures describing the percentage of residents who (i) are Hispanic; (ii) are foreign-born Hispanic; (iii) speak Spanish and limited English; and (iv) live in linguistically isolated households speaking Spanish, i.e., Spanish is the primary language spoken in the household and no member speaks English “very well” or is proficient in English. We categorized enclave scores into statewide quintiles to identify the least (Q1) and most culturally/ethnically distinct neighborhoods (Q5). We classified tracts as enclaves if they were in (i) Q5 with >250 Hispanic residents or (ii) Q4 with >250 Hispanic residents and spatially adjacent to a Q5 tract.

### Covariates

We included the following demographic, clinical, and treatment factors as covariates: sex, age at diagnosis in years, race/ethnicity (Hispanic, non-Hispanic Asian/Pacific Islander, non-Hispanic Black, non-Hispanic White, other non-Hispanic, or missing), National Cancer Institute (NCI) risk group at diagnosis (standard risk, high risk), ALL immunophenotype and cytogenetic subtypes, and treating institutions. Cytogenetic subtypes were classified based on prognostic groupings defined by Children’s Oncology Group (COG), the largest pediatric cancer cooperative group in North America. The COG defines favorable cytogenetics as *ETV6::RUNX1* and double trisomies of chromosomes 4 and 10. Unfavorable subtypes include *BCR::ABL1* (Philadelphia chromosome positive), hypodiploidy (<45 chromosomes), intrachromosomal amplification of chromosome 21, and *KMT2A* rearrangements. Other subtypes were classified as neutral. Cases with incomplete cytogenetic data were categorized as missing (33).

### Statistical analysis

We described the distribution of variables for overall study population and by site using counts and percentages. To address the generalizability of our sample compared with population-based data, we compared the descriptive characteristics of patients included in our analysis with those from the population-based Texas Cancer Registry (TCR) which captures pediatric ALL cases statewide using similar diagnosis years (2005–2011) using *χ*^2^ tests. Furthermore, we also compared demographic and clinical characteristics of patients in our study analytic sample with the complete REDIAL Consortium cohort using *χ*^2^ tests.

We evaluated associations of nSES and Hispanic enclave residence with MRD positivity and relapse using crude and adjusted logistic regression models. Covariates were selected *a priori* based on literature review and included sex, age at diagnosis, race/ethnicity, cytogenetic features, NCI risk group at diagnosis, ALL immunophenotype, and treating institutions.

We summarized survival time using the median and interquartile range (IQR) in years and median of Kaplan–Meier (KM) survival estimate for OS and EFS. We plotted KM curves to compare OS and EFS by enclave and nSES categories. We estimated hazard ratio (HR) and 95% confidence interval (CI) of mortality using Cox proportional hazards regression. We evaluated proportional hazards assumption (PHA) using (i) visual inspection of log[−log(survival)] curves versus log of survival time and (ii) inclusion of interaction terms between covariates and a function of time (i.e., time-dependent covariates included to assess whether the effect of a covariate changed over time) in the Cox model. We found no evidence of PHA violation. We used robust (sandwich) standard errors in both logistic and Cox regression models to account for within–census tract correlation.

### Subgroup analysis

Because the health effects of neighborhood-level deprivation may vary by race and ethnicity (34, 35) and because Hispanic children have poorer outcomes following diagnosis with ALL (15–20, 36), we performed subgroup analysis among Hispanic children.

### Sensitivity analysis

Our team previously showed that low nSES (13) and Hispanic enclave status (12) were associated with poor OS in children with ALL using the population-based TCR, which does not capture detailed clinical and molecular data. Clinical and molecular prognostic factors were included because they are strongly associated with pediatric ALL treatment response and survival (37). Our primary analytic objective is to evaluate whether neighborhood measures were associated with outcomes beyond established disease-risk characteristics and treatment factors. Because the causal relationships among neighborhood context, disease biology, and treatment outcomes are complex and potentially interrelated, we additionally performed a sensitivity analysis restricting covariate adjustment to variables available in the TCR (i.e., sex, race/ethnicity, and age at diagnosis) to more closely approximate existing TCR-based models and determine whether inclusion of additional clinical and molecular information altered our findings.

## Results

### Characteristics

The study sample included 1,348 Texas children, adolescents, and young adults diagnosed with ALL at 0 to 24 years of age between 2005 and 2017. Among these, 41% (*n* = 550) resided in tracts with low nSES and 42% (*n* = 560) resided in a Hispanic enclave at the time of their diagnosis. Study participants were predominantly male (56%), Hispanic (59%), diagnosed with B-cell ALL (B-ALL; 90%), and enrolled on a clinical trial (55%). Table 1 presents characteristics according to study sites. Supplementary Figure S1 shows the geographic distribution of nSES and Hispanic enclaves across Texas from 2000 to 2016. Hispanic enclaves remained heavily concentrated in the border region and within the largest metro areas, including Austin, Houston, and Dallas–Fort Worth metropolitan areas. Low-nSES areas were consistently concentrated in South Texas and rural East Texas across all three time points, with some presence in West Texas regions (e.g., El Paso).

**Table 1.** Demographic and clinical characteristics of children treated for ALL between 2005 and 2017, overall and by treating institutions within the REDIAL Consortium.

Characteristic	Study sample, *N* (%) (*N* = 1,348)	TCH, *N* (%) (*N* = 853)	Cook, *N* (%) (*N* = 273)	Vannie Cook, *N* (%) (*N* = 222)
Sex	​	​	​	​
Female	592 (43.9)	383 (44.9)	122 (44.7)	87 (39.2)
Male	756 (56.1)	470 (55.1)	151 (55.3)	135 (60.8)
Age at diagnosis (years)	​	​	​	​
<5	593 (44)	371 (43.5)	128 (46.9)	94 (42.3)
5–9	372 (27.6)	241 (28.3)	66 (24.2)	65 (29.3)
≥10	383 (28.4)	241 (28.3)	79 (28.9)	63 (28.4)
Race/ethnicity	​	​	​	​
Hispanic	801 (59.4)	465 (54.5)	121 (44.3)	215 (96.8)
Non-Hispanic Asian	59 (4.4)	54 (6.3)	3 (1.1)	2 (2.9)
Non-Hispanic Black	81 (6)	66 (7.7)	15 (5.5)	0 (0)
Non-Hispanic White	389 (28.9)	256 (30)	129 (47.3)	4 (1.8)
Other non-Hispanic	8 (0.6)	5 (0.6)	3 (1.1)	0 (0)
Missing	10 (0.7)	7 (0.8)	2 (0.7)	1 (0.5)
ALL cytogenetic subtypes	​	​	​	​
Favorable	489 (36.3)	314 (36.8)	94 (34.4)	81 (36.5)
Unfavorable	139 (10.3)	101 (11.8)	25 (9.2)	13 (5.9)
Not prognostic	452 (33.5)	340 (39.9)	1 (0.4)	111 (50)
Missing	268 (19.9)	98 (11.5)	153 (56)	17 (7.7)
NCI risk group	​	​	​	​
Standard	746 (55.3)	455 (53.3)	156 (57.1)	135 (60.8)
High	570 (42.3)	374 (43.8)	113 (41.4)	83 (37.4)
Missing	32 (2.4)	24 (2.8)	4 (1.5)	4 (1.8)
ALL immunophenotype	​	​	​	​
B-cell ALL	1,206 (89.5)	765 (89.7)	237 (86.8)	204 (91.9)
T-cell ALL	140 (10.4)	88 (10.3)	34 (12.5)	18 (8.1)
Missing	2 (0.2)	0 (0)	2 (0.7)	0 (0)
Clinical trial enrollment	​	​	​	​
Enrolled	734 (54.5)	533 (62.5)	184 (67.4)	17 (7.7)
Not enrolled	602 (44.7)	313 (36.7)	87 (31.9)	202 (91)
Missing	12 (0.9)	7 (0.8)	2 (0.7)	3 (1.4)
Enclave status	​	​	​	​
Enclave	560 (41.5)	305 (35.8)	53 (19.4)	202 (91)
Non-enclave	693 (51.4)	478 (56)	213 (78)	2 (0.9)
Missing	95 (7.1)	70 (8.2)	7 (2.6)	18 (8.1)
nSES	​	​	​	​
Low (Yost Q1, Q2)	550 (40.8)	297 (34.8)	90 (33)	163 (73.4)
High (Yost Q3–Q5)	703 (52.2)	486 (57)	176 (64.5)	41 (18.5)
Missing	95 (7.1)	70 (8.2)	7 (2.6)	18 (8.1)
EOI MRD	​	​	​	​
Negative	990 (73.4)	618 (72.5)	209 (76.6)	163 (73.4)
Positive	288 (21.4)	187 (21.9)	54 (19.8)	47 (21.2)
Missing	70 (5.2)	48 (5.6)	10 (3.7)	12 (5.4)
Relapse	​	​	​	​
No	1,169 (86.7)	743 (87.1)	242 (88.6)	184 (82.9)
Yes	179 (13.3)	110 (12.9)	31 (11.4)	38 (17.1)
Vital status	​	​	​	​
Alive	1,221 (90.6)	764 (89.6)	256 (93.8)	201 (90.5)
Deceased	127 (9.4)	89 (10.4)	17 (6.2)	21 (9.5)
Events (relapse, death)	​	​	​	​
No	1,125 (83.5)	711 (83.4)	234 (85.7)	180 (81.1)
Yes	223 (16.5)	142 (16.7)	39 (14.3)	42 (18.9)
Median (IQR) follow-up time for OS (years)	5.6 (3.4–8.1)	6.1 (3.8–9.2)	4.4 (2.7–6.6)	5 (3–7.7)
Median (IQR) follow-up time for EFS (years)	5.2 (3.1–7.9)	5.8 (3.5–8.8)	4.1 (2.5–6.6)	4.4 (2.8–7.3)

Compared with the statewide TCR population of children diagnosed with ALL during a similar period, our REDIAL analytic sample was similar with respect to sex and age at diagnosis (Supplementary Table S1). The REDIAL sample included a slightly higher proportion of Hispanic children (59.4% vs. 55.2%) and a lower proportion of non-Hispanic White children (28.9% vs. 33.3%). Additionally, our analytic sample was similar to the complete REDIAL Consortium cohort (Supplementary Table S2); however, the analytic sample included a slightly higher proportion of Hispanic children (59.4% vs. 56.4%) and had a lower prevalence of EOI MRD positivity (21.4% vs. 25%).

**Figure 1.**
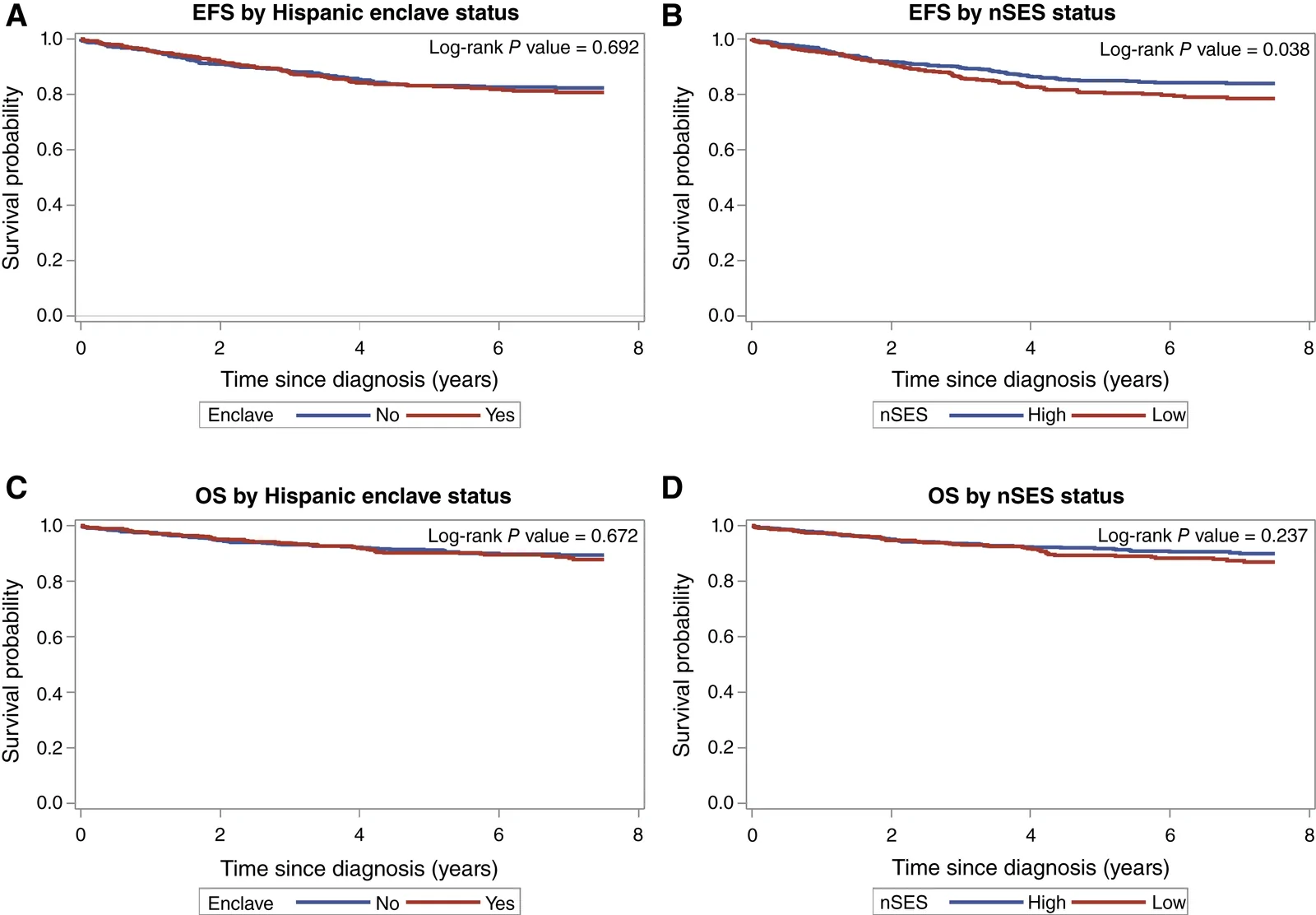
KM analysis of EFS and OS by Hispanic enclave status (**A** and **C**) and nSES (**B** and **D**).

The median follow-up times were 5.2 (IQR, 3.1–7.9) and 5.6 (IQR, 3.4–8.1) years for OS and EFS, respectively (Table 1). KM analysis indicated poorer 5-year EFS for children living in low-nSES areas compared with those living in high-nSES areas (81% vs. 85%; *P* = 0.04; Fig. 1).

### EOI MRD positivity

In multivariable logistic regression models adjusted for demographic and clinical features and treating institutions, Hispanic enclave residence was not associated with MRD positivity [adjusted odds ratio (aOR), 1.22; 95% CI, 0.82–1.80]. Subgroup analysis restricted to Hispanic children with ALL yielded similar results (aOR, 1.15; 95% CI, 0.74–1.79; Table 2). Similarly, low nSES was not associated with MRD positivity either in overall sample (aOR, 1.06; 95% CI, 0.73–1.55) or among Hispanic children (aOR, 0.93; 95% CI, 0.61–1.41). Sensitivity analysis limiting to covariates available for the TCR produced similar null associations for both Hispanic enclaves (aOR, 1.13; 95% CI, 0.82–1.55) and low nSES (aOR, 1.05; 95% CI, 0.78–1.41; Table 2).

**Table 2.** ORs and 95% CIs of EOI MRD positivity and relapse among children living in low-nSES areas or Hispanic enclaves.

Neighborhood feature	EOI MRD positivity		Relapse	
	cOR (95% CI)	aOR (95% CI)^a^	cOR (95% CI)	aOR (95% CI)^a^
Main analyses				
Enclave status	​	​	​	​
Enclave	1.24 (0.95–1.61)	1.23 (0.83–1.82)	1.16 (0.82–1.63)	0.88 (0.52–1.49)
Non-enclave	1	1	1	1
nSES	​	​	​	​
Low (Yost Q1, Q2)	1.21 (0.93–1.58)	1.09 (0.75–1.58)	1.36 (0.98–1.90)	1.04 (0.66–1.64)
High (Yost Q3–Q5)	1	1	1	1
Subgroup analyses among Hispanic children				
Enclave status	​	​	​	​
Enclave	1.07 (0.75–1.52)	1.18 (0.75–1.85)	0.91 (0.59–1.40)	0.79 (0.45–1.42)
Non-enclave	1	1	1	1
nSES	​	​	​	​
Low (Yost Q1, Q2)	1.01 (0.72–1.41)	0.97 (0.64–1.48)	1.17 (0.77–1.78)	1.11 (0.66–1.87)
High (Yost Q3–Q5)	1	1	1	1
Sensitivity analyses using TCR covariates^b^				
Enclave status	​	​	​	​
Enclave	​	1.14 (0.83–1.56)	​	0.94 (0.63–1.40)
Non-enclave	​	1	​	1
nSES	​	​	​	​
Low (Yost Q1, Q2)	​	1.04 (0.77–1.40)	​	1.10 (0.76–1.58)
High (Yost Q3–Q5)	​	1	​	1

Abbreviation: cOR, crude OR.

a Adjusted models for main and subgroup analyses include the following covariates: sex, age at diagnosis, race/ethnicity, cytogenetic features, NCI risk group at diagnosis, ALL immunophenotype, and treating institutions.

b Adjusted models for sensitivity analyses include the following covariates: sex, age at diagnosis, and race/ethnicity.

### Relapse

Hispanic enclave residence was not associated with relapse in adjusted models (aOR, 0.88; 95% CI, 0.52–1.49). Subgroup analyses among Hispanic children (aOR, 0.79; 95% CI, 0.45–1.42) and sensitivity analysis using TCR-available covariates (aOR, 0.94; 95% CI, 0.63–1.40) yielded similar results (Table 2). Similarly, no association was observed between low nSES and relapse in overall cohort (aOR, 1.04; 95% CI, 0.66–1.64) or among Hispanic children (aOR, 1.11; 95% CI, 0.66–1.87). Sensitivity analysis limited to TCR-available covariates produced similar findings (aOR, 1.10; 95% CI, 0.76–1.58; Table 2).

### EFS

Hispanic enclave residence was not associated with EFS in adjusted Cox models [adjusted HR (aHR), 0.81; 95% CI, 0.52–1.26], including analysis restricted to Hispanic children (aHR, 0.78; 95% CI, 0.48–1.27) and models using TCR-available covariates (aHR for EFS, 0.88; 95% CI, 0.63–1.22; Table 3). In unadjusted analyses, low nSES was associated with worse EFS (crude HR, 1.33; 95% CI, 1.01–1.76); however, this association was attenuated after adjusting for covariates (aHR, 1.04; 95% CI, 0.71–1.54). Among Hispanic children, low nSES was not associated with EFS (aHR, 1.07; 95% CI, 0.69–1.67), and findings remained consistent in TCR-adjusted models without clinical and molecular covariates (aHR, 1.10; 95% CI, 0.81–1.48; Table 3).

**Table 3.** Cox proportional hazard regression analyses for associations of Hispanic enclave status and nSES with OS and EFS.

​Neighborhood feature	EFS		OS	
	cHR (95% CI)	aHR (95% CI)^a^	cHR (95% CI)	aHR (95% CI)^a^
Enclave status	​	​	​	​
Enclave	1.05 (0.79–1.40)	0.81 (0.52–1.26)	1.07 (0.75–1.51)	1.07 (0.59–1.94)
Non-enclave	1	1	1	1
nSES	​	​	​	​
Low (Yost Q1, Q2)	**1.33 (1.01–1.76)**	1.04 (0.71–1.54)	1.23 (0.87–1.73)	0.89 (0.53–1.51)
High (Yost Q3–Q5)	1	1	1	1
Subgroup analyses among Hispanic children				
Enclave status	​	​	​	​
Enclave	0.80 (0.56–1.13)	0.78 (0.48–1.27)	0.84 (0.53–1.33)	0.94 (0.50–1.78)
Non-enclave	1	1	1	1
nSES	​	​	​	​
Low (Yost Q1, Q2)	1.18 (0.84–1.67)	1.07 (0.69–1.67)	1.30 (0.82–2.07)	0.93 (0.51–1.70)
High (Yost Q3–Q5)	1	1	1	1
Sensitivity analyses using TCR covariates				
Enclave status	​	​	​	​
Enclave	​	0.88 (0.63–1.22)	​	1.01 (0.67–1.54)
Non-enclave	​	1	​	1
nSES	​	​	​	​
Low (Yost Q1, Q2)	​	1.10 (0.81–1.48)	​	1.05 (0.72–1.54)
High (Yost Q3–Q5)	​	1	​	1

Abbreviation: cHR, crude HR.

Bold values are statistically significant at *P* < 0.05.

a Adjusted models for main and subgroup analyses include the following covariates: sex, age at diagnosis, race/ethnicity, EOI MRD, cytogenetic features, NCI risk group at diagnosis, ALL immunophenotype, and treating institutions. Adjusted models for sensitivity analyses include the following covariates: sex, age at diagnosis, and race/ethnicity.

### OS

In adjusted Cox models, Hispanic enclave residence was not associated with mortality (aHR, 1.07; 95% CI, 0.59–1.94). Results remained consistent in analysis limited to Hispanic children (aHR, 0.94; 95% CI, 0.50–1.78) and in models using TCR-available covariates (aHR, 1.01; 95% CI, 0.67–1.54; Table 3). Similarly, low nSES was not associated with OS in the overall cohort (aHR, 0.89; 95% CI, 0.53–1.51), among Hispanic children (aHR, 0.93; 95% CI, 0.51–1.70), or when restricted to TCR-available covariates (aHR, 1.05; 95% CI, 0.72–1.54; Table 3).

## Discussion

Racial, ethnic, and socioeconomic disparities in treatment outcomes have been described for children with ALL. The underlying mechanisms have not been fully characterized but may include differences in access to care, treatment adherence, and disease biology—factors that may be influenced by residential environments. This study examined the associations among nSES, Hispanic enclave residence, and treatment outcomes in pediatric ALL. Despite prior reports that neighborhood-level factors may be associated with treatment response and survival, we did not find evidence that low nSES or residence in a Hispanic enclave was associated with EOI MRD positivity, relapse, or death in children with ALL after adjusting for clinical and demographic factors. These findings suggest that the composite neighborhood measures evaluated in this study were not independently associated with pediatric ALL outcomes within this cohort.

The role of Hispanic enclaves in health outcomes is complex because enclaves can have both positive and adverse health effects on their residents (38). Although enclaves may convey economic hardship and cultural isolation that can limit access to care (25), they may also provide strong social support that facilitates care adherence (9). Studies on the role of enclaves in cancer outcomes among adults have reported mixed findings. Hispanic enclave residence has been associated with survival advantages in colon (39) and prostate (40) cancers. However, findings from breast cancer are inconsistent, and no association was observed in a study about endometrial cancer (41). In pediatric cancer, limited evidence exists. A recent single-center study from TCH found that higher Latinx enclave index scores were associated with MRD positivity, with children residing in areas with the highest enclave index scores showing the greatest odds of MRD positivity (42). However, our present study did not observe such associations. This difference may partly reflect variation in study design and populations. Our study included a larger and more diverse sample across multiple institutions, whereas the above aforementioned study focused on a single-center cohort, purposefully enriched for MRD-positive patients. Notably, our article is among the first to evaluate enclaves in pediatric cancer, and we found no evidence of either protective or adverse effects. Possibly, mechanisms conferring benefit to adults, such as social cohesion or access to culturally aligned resources (40, 43–45), may operate differently for children, thereby explaining lack of association in our analysis. Alternatively, more standardized treatment protocols for children compared with adults may buffer potential negative impacts on outcomes.

Socioeconomic disadvantage has been linked to poorer outcomes in adolescent and young adults with ALL (10, 28, 46, 47), but its impact in younger children remains less clear. Differences between children and adults may stem from both biological and treatment-related factors, with most research on nSES and cancer health disparities conducted in adults. Children with ALL often present with more favorable cytogenetic features, whereas adults more commonly exhibit high-risk cytogenetic features. Additionally, children tend to tolerate intensive chemotherapy regimens better than adults and have greater access to standardized treatment protocols and opportunity for clinical trial enrollment compared with adults, which may buffer the impact of neighborhood disadvantage on treatment response and survival (48). In contrast, adults may face prolonged exposure to socioeconomic stressors, including economic hardship and barriers to healthcare access, which can uniquely affect treatment response and survival. These differences likely contribute to the inconsistent findings on nSES and ALL outcomes across pediatric and adult populations.

Our results contrast with registry-based studies that reported poorer OS among children with ALL residing in socioeconomically disadvantaged neighborhoods (13) and Hispanic enclaves at the time of their diagnosis (12). To facilitate comparison with prior TCR analyses, we additionally fit reduced models using only demographic covariates available in the TCR. Findings remained null, suggesting that differences between studies may not be solely attributable to adjustment for detailed clinical and molecular characteristics. Therefore, these differences may reflect variations in study design, cohort composition, geographic distribution, referral patterns, and neighborhood exposure measurement. For example, prior research using the TCR included all Texas children diagnosed with ALL between 1995 and 2011, whereas our present study examined cases diagnosed from 2005 to 2017 at three large pediatric oncology centers within the REDIAL Consortium. Compared with studies using TCR data, our sample may be more homogeneous in regard to place of residence, demographics, or access to care, which may have limited our ability to detect associations. Additionally, the prior studies using TCR measured nSES using the Area Deprivation Index (ADI), whereas we used the Yost socioeconomic index. Although both are multidimensional neighborhood measures, they capture different constructs. Compared with the Yost index, the ADI captures a broader range of deprivation-related indicators (e.g., poverty, housing quality, and employment) and is more sensitive to missing or extreme values (49). Unlike the ADI, the Yost index incorporates education, income, and occupational characteristics commonly used in cancer disparity research. These methodologic differences in the estimation of nSES may partly contribute to differences in findings; however, our null findings were robust when substituting the ADI for the Yost index (Supplementary Tables S3 and S4). Notably, our prior work observed a decline in HR for mortality among children in disadvantaged neighborhoods in the later years of study period (2004–2011) which closely aligns with the current study time period (2005–2017), suggesting that continued improvements in therapy and supportive care could be narrowing socioeconomic disparities (50, 51). Unlike previous TCR analyses, the present study accounted for ALL cytogenetic subtype, which varied by nSES and may therefore contribute to the variation in findings. We found that children from low nSES had higher odds of “not prognostic” cytogenetics (vs. “favorable”; OR, 1.56; 95% CI, 1.20–2.04) compared with children from high nSES. This finding should be interpreted with caution, as *CRLF2* rearrangements were not assessed for most participants (20%) in this cohort and comprise a subset of “not prognostic” category (35%). Given that *CRLF2* alterations are more prevalent among Hispanic children and have been associated with poorer outcomes (52), the lack of these data may partially obscure underlying sociodemographic differences in cytogenetic risk profiles. For all these reasons, our findings should be interpreted within the context of this specific multicenter cohort rather than as definitive evidence against neighborhood effects in pediatric ALL.

Evidence from other regions further underscores how neighborhood classification methods and geographic context shape findings. A California-based study of children, adolescents, and young adults with ALL found higher mortality risk among children living in mixed SES suburbs and Hispanic small towns compared with those in upper middle-class suburban neighborhoods (10). That study used latent class analysis to identify multidimensional neighborhood archetypes, incorporating socioeconomic composition, demographics, residential mobility, and food environment. Their findings highlight that children in mixed or lower SES environments and higher concentrations of racial/ethnic minorities experienced poorer OS, independent of race, ethnicity, insurance, or treatment facility. By contrast, a Canadian multicenter study using income quintiles found no overall association between neighborhood income and OS, although children in the highest quintile had significantly superior EFS than those in the lowest quintile (11). Similarly, a large retrospective COG analysis of patients with relapsed ALL observed differences in post-relapse OS in B-ALL by Zone Improvement Plan code–based income; children from the highest-income areas had superior 5-year OS compared with those from the lowest (53). Taken together, these findings highlight the potential sensitivity of results to neighborhood definitions, outcome measures, local context, and healthcare systems and suggest that although some studies observe disparities, it remains important to consider the broader ways in which neighborhood context may influence pediatric ALL survival, both directly and through intermediary factors. Moreover, limited variability in exposures or outcomes across study populations may reduce power to detect associations, even when nSES exerts meaningful effects.

This study has several strengths, most notably detailed clinical and demographic data from a large, diverse sample of children with ALL. Some limitations should also be considered. Although we accounted for key clinical and demographic confounders, neighborhood factors could affect ALL outcomes through unmeasured individual or neighborhood mechanisms including but not limited to treatment adherence, delays in supportive care utilization, caregiver stress, and residential mobility or instability. Approximately 40% of participants resided in low nSES or Hispanic enclaves, which may have limited statistical power to detect small-to-moderate associations with the outcomes we evaluated. Additionally, residential address data were available for only three REDIAL centers; however, comparison of characteristics with the complete REDIAL cohort suggested limited evidence of selection bias (Supplementary Table S2). Furthermore, a comparison of our analytic sample with statewide data from the TCR (Supplementary Table S1) suggests modest differences in race/ethnicity and vital status. Our study population may not be representative of all Texas children diagnosed with ALL. Lastly, our study focused on early treatment response and survival outcomes, leaving open clinically relevant questions about acute toxicities, and long-term impact of neighborhood factors on cancer survivorship, late effects, and quality of life, which warrant further investigation.

In conclusion, our findings do not suggest that nSES and Hispanic enclave residence are associated with EOI MRD, relapse, or death due to pediatric ALL in this sample. Although neighborhood disparities in some pediatric ALL outcomes are reported in other settings, our findings suggest that in the context of standardized treatment with high rates of clinical trial participation, neighborhood-level factors may exert minimal influence on clinical outcomes in pediatric ALL. These apparently conflicting findings may reflect differences in cohort composition, treatment setting, neighborhood exposure definitions, or temporal improvements in pediatric ALL care, and our report should not be interpreted as evidence that neighborhood context is universally unrelated to pediatric ALL outcomes. Future studies should explore other social determinants and explore whether neighborhood context affects disease characteristics, survivorship, and late effects to fully understand how social environments shape the lives of childhood ALL survivors.

## Supplementary Material

Supplementary Table S1 Demographic characteristics of children diagnosed with ALL within the REDIAL Consortium (2005-2017) and those children residing in Texas based on data obtained from Texas Cancer Registry (TCR) (2005-2011).

Supplementary Table S2 Comparison of demographic and clinical characteristics of children treated for ALL between 2005-2017 between our study sample and complete REDIAL Consortium.

Supplementary Table S3 Odds ratio and 95% confidence interval of end-induction minimal residual disease positivity and relapse according to census trat-level Area Deprivation Index score.

Supplementary Table S4 Cox proportional hazards regression models for the association of census tract-level Area Deprivation Index score with overall and event-free survival.

Supplementary Figure S1 Distribution of Hispanic enclave index and Yost index in Texas using 2000 decennial US census data, ACS 2008-2012 and ACS 2012-2016.

## Data Availability

Because the study was conducted using retrospectively collected data from electronic health records, we were unable to obtain written informed consent from participants for data to be shared publicly. Study data are available upon request to the REDIAL investigators (K.R. Rabin, P.J. Lupo, and M.E. Scheurer).
