# Supplementary material for: Neighborhood Factors and Treatment Outcomes in Pediatric Acute Lymphoblastic Leukemia: A REDIAL Consortium Report
Source: Cancer Res Commun. 2026 Sep 4;6(9):2079–89. doi: 10.1158/2767-9764.CRC-26-0134 (PMC13542063; doi:10.1158/2767-9764.CRC-26-0134)
Supplement: Supplementary file 1 [file crc-26-0134_supplementary_figure_s1_suppsf1.docx]

**Supplementary figure 1: Distribution of Hispanic enclave index and Yost index in Texas using 2000 decennial US census data, ACS 2008-2012 and ACS 2012-2016.**
