# Supplementary material for: Neighborhood Factors and Treatment Outcomes in Pediatric Acute Lymphoblastic Leukemia: A REDIAL Consortium Report
Source: Cancer Res Commun. 2026 Sep 4;6(9):2079–89. doi: 10.1158/2767-9764.CRC-26-0134 (PMC13542063; doi:10.1158/2767-9764.CRC-26-0134)
Supplement: Supplementary file 2 [file crc-26-0134_supplementary_table_s1_suppst1.docx]

**Supplementary Table 1: Demographic characteristics of children diagnosed with ALL within the REDIAL Consortium (2005-2017) and those children residing in Texas based on data obtained from Texas Cancer Registry (TCR) (2005-2011).**

|  | **REDIAL sample, n (%)** | **TCR, N (%)^a^** | **P-value** |
| --- | --- | --- | --- |
| **Sex** |  |  | 0.96 |
| Female | 592 (43.9) | 838 (44) |  |
| Male | 756 (56.1) | 1066 (56) |  |
| **Age at diagnosis (years)** |  |  | 0.65 |
| <5 | 593 (44.0) | 837 (45.5) |  |
| 5-9 | 372 (27.6) | 485 (26.4) |  |
| ≥10 | 383 (28.4) | 518 (28.2) |  |
| **Race/ethnicity** |  |  | 0.03 |
| Hispanic | 801 (59.4) | 1050 (55.2) |  |
| Non-Hispanic Black | 81 (6) | 127 (6.7) |  |
| Non-Hispanic White | 389 (28.9) | 633 (33.3) |  |
| Other non-Hispanic/Unknown | 77 (5.7) | 94 (4.9) |  |
| **Vital status** |  |  | <0.01 |
| Alive | 1221 (90.6) | 1661 (87.2) |  |
| Deceased | 127 (9.4) | 243 (12.8) |  |

^a^ Values representing the TCR population corresponds to ALL patients diagnosed between 2005 and 2011. The P-value reflects chi-square tests comparing the REDIAL sample with TCR ALL cases.
