# Supplementary material for: Neighborhood Factors and Treatment Outcomes in Pediatric Acute Lymphoblastic Leukemia: A REDIAL Consortium Report
Source: Cancer Res Commun. 2026 Sep 4;6(9):2079–89. doi: 10.1158/2767-9764.CRC-26-0134 (PMC13542063; doi:10.1158/2767-9764.CRC-26-0134)
Supplement: Supplementary file 3 [file crc-26-0134_supplementary_table_s2_suppst2.docx]

**Supplementary Table 2: Comparison of demographic and clinical characteristics** **of children treated for ALL between 2005-2017 between our study sample and complete REDIAL Consortium.**

|  | **Subsample, n (%)** | **Complete cohort, N (%)** | **P-value** |
| --- | --- | --- | --- |
| **Sex** |  |  | 0.61 |
| Female | 592 (43.9) | 915 (43.0) |  |
| Male | 756 (56.1) | 1211 (57.0) |  |
| **Age at diagnosis (years)** |  |  | 0.64 |
| <5 | 593 (44.0) | 970 (45.6) |  |
| 5-9 | 372 (27.6) | 571 (26.9) |  |
| ≥10 | 383 (28.4) | 585 (27.5) |  |
| **Race/ethnicity** |  |  | 0.02 |
| Hispanic | 801 (59.4) | 1199 (56.4) |  |
| Non-Hispanic Asian | 59 (4.4) | 96 (4.5) |  |
| Non-Hispanic Black | 81 (6.0) | 131 (6.2) |  |
| Non-Hispanic White | 389 (28.9) | 636 (29.9) |  |
| Other non-Hispanic | 8 (0.6) | 40 (1.9) |  |
| Missing | 10 (0.7) | 24 (1.1) |  |
| **ALL cytogenetics** |  |  | 0.02 |
| Favorable | 489 (36.3) | 770 (36.2) |  |
| Unfavorable | 139 (10.3) | 184 (8.7) |  |
| Not prognostic | 452 (33.5) | 804 (37.8) |  |
| Missing | 268 (19.9) | 368 (17.3) |  |
| **NCI risk group** |  |  | 0.12 |
| Standard | 746 (55.3) | 1119 (52.6) |  |
| High | 570 (42.3) | 954 (44.9) |  |
| Missing | 32 (2.4) | 53 (2.5) |  |
| **ALL immunophenotype** |  |  | 0.78 |
| B-ALL | 1206 (89.5) | 1911 (89.9) |  |
| T-ALL | 140 (10.4) | 215 (10.1) |  |
| Missing | 2 (0.2) |  |  |
| **Clinical trial enrollment** |  |  | 0.90 |
| Enrolled | 734 (54.5) | 1142 (53.72) |  |
| Not enrolled | 602 (44.7) | 928 (43.7) |  |
| Missing | 12 (0.9) | 56 (2.6) |  |
| **Enclave status** |  |  | 0.10 |
| Enclave | 560 (41.5) | 730 (34.3) |  |
| Non-enclave | 693 (51.4) | 1023 (48.1) |  |
| Missing | 95 (7.1) | 373 (17.5) |  |
| **Neighborhood SES** |  |  | 0.15 |
| Low (Yost Q1, Q2) | 550 (40.8) | 723 (34) |  |
| High (Yost Q3-Q5) | 703 (52.2) | 1030 (48.5) |  |
| Missing | 95 (7.1) | 373 (17.5) |  |
| **End-induction MRD** |  |  | 0.002 |
| Negative | 990 (73.4) | 1415 (66.6) |  |
| Positive | 288 (21.4) | 532 (25.0) |  |
| Missing | 70 (5.2) | 179 (8.4) |  |
| **Relapse** | 179 (13.3) | - |  |
| **Vital status** |  |  | 0.89 |
| Alive | 1221 (90.6) | 1894 (89.1) |  |
| Deceased | 127 (9.4) | 194 (9.1) |  |
| Missing | - | 38 (1.8) |  |
