# Supplementary material for: Neighborhood Factors and Treatment Outcomes in Pediatric Acute Lymphoblastic Leukemia: A REDIAL Consortium Report
Source: Cancer Res Commun. 2026 Sep 4;6(9):2079–89. doi: 10.1158/2767-9764.CRC-26-0134 (PMC13542063; doi:10.1158/2767-9764.CRC-26-0134)
Supplement: Supplementary file 4 [file crc-26-0134_supplementary_table_s3_suppst3.docx]

**Supplementary Table 3: Odds ratio and 95% confidence interval of end-induction minimal residual disease positivity and relapse according to census trat-level Area Deprivation Index score.**

|  | **EOI MRD positivity** | | **Relapse** | |
| --- | --- | --- | --- | --- |
| **ADI quartile** | **cOR (95% CI)** | **aOR (95% CI)^a^** | **cOR (95% CI)** | **aOR (95% CI)^a^** |
| **Main analyses** | | | | |
| Q1 (least disadvantaged) | 1.00 | 1.00 | 1.00 | 1.00 |
| Q2 | 1.18 (0.79, 1.78) | 1.15 (0.69, 1.91) | 0.93 (0.56, 1.54) | 0.85 (0.45, 1.61) |
| Q3 | 1.38 (0.93, 2.04) | 1.11 (0.67, 1.85) | 0.97 (0.60, 1.59) | 0.70 (0.37, 1.33) |
| Q4 (most disadvantaged) | 1.33 (0.93, 1.92) | 1.14 (0.69, 1.89) | 1.41 (0.92, 2.17) | 1.04 (0.58, 1.89) |
| **Subgroup analyses among Hispanic children** | | | | |
| Q1 (least disadvantaged) | 1.00 | 1.00 | 1.00 | 1.00 |
| Q2 | 1.09 (0.58, 2.05) | 1.14 (0.53, 2.45) | 1.09 (0.52, 2.29) | 0.93 (0.38, 2.28) |
| Q3 | 1.10 (0.62, 1.96) | 0.94 (0.47, 1.88) | 1.01 (0.51, 2.00) | 0.80 (0.36, 1.80) |
| Q4 (most disadvantaged) | 1.04 (0.61, 1.77) | 1.03 (0.54, 1.98) | 1.10 (0.59, 2.04) | 0.98 (0.46, 2.07) |
| **Sensitivity analyses using TCR covariates^b^** | | | | |
| Q1 (least disadvantaged) | - | 1.00 | - | 1.00 |
| Q2 | - | 1.20 (0.79, 1.82) | - | 0.89 (0.52, 1.50) |
| Q3 | - | 1.22 (0.80, 1.85) | - | 0.79 (0.47, 1.33) |
| Q4 (most disadvantaged) | - | 1.17 (0.77, 1.76) | - | 1.05 (0.65, 1.71) |

Abbreviations: ADI=Area Deprivation Index; aOR=adjusted hazard ratio; cOR=crude hazard ratio; CI=confidence interval; EOI MRD=end-of-induction minimal residual disease; nSES=neighborhood socioeconomic status; TCR=Texas Cancer Registry.

^a^ Adjusted for sex, age at diagnosis, race/ethnicity, cytogenetic features, National Cancer Institute risk group at diagnosis, immunophenotype, and treating institution.

^b^ Adjusted for sex, age at diagnosis, and race/ethnicity.
