# Supplementary material for: Neighborhood Factors and Treatment Outcomes in Pediatric Acute Lymphoblastic Leukemia: A REDIAL Consortium Report
Source: Cancer Res Commun. 2026 Sep 4;6(9):2079–89. doi: 10.1158/2767-9764.CRC-26-0134 (PMC13542063; doi:10.1158/2767-9764.CRC-26-0134)
Supplement: Supplementary file 5 [file crc-26-0134_supplementary_table_s4_suppst4.docx]

**Supplementary Table 4: Cox proportional hazards regression models for the association of census tract-level Area Deprivation Index score with overall and event-free survival.**

|  | **Event-free survival** | | **Overall survival** | |
| --- | --- | --- | --- | --- |
| **ADI quartile** | **cHR (95% CI)** | **aHR (95% CI)^a^** | **cHR (95% CI)** | **aHR (95% CI)^a^** |
| **Main analyses** | | | | |
| Q1 (least disadvantaged) | 1.00 | 1.00 | 1.00 | 1.00 |
| Q2 | 0.91 (0.59, 1.39) | 0.94 (0.56, 1.58) | 0.78 (0.45, 1.36) | 1.02 (0.53, 1.96) |
| Q3 | 1.04 (0.69, 1.56) | 0.93 (0.57, 1.54) | 0.97 (0.58, 1.62) | 1.08 (0.57, 2.03) |
| Q4 (most disadvantaged) | 1.30 (0.91, 1.85) | 1.09 (0.68, 1.74) | 1.05 (0.66, 1.67) | 0.95 (0.51, 1.78) |
| **Subgroup analyses among Hispanic children** | | | | |
| Q1 (least disadvantaged) | 1.00 | 1.00 | 1.00 | 1.00 |
| Q2 | 0.89 (0.48, 1.65) | 0.87 (0.42, 1.80) | 0.80 (0.34, 1.88) | 0.98 (0.38, 2.49) |
| Q3 | 1.05 (0.60, 1.81) | 0.95 (0.51, 1.76) | 1.10 (0.53, 2.29) | 0.98 (0.43, 2.25) |
| Q4 (most disadvantaged) | 1.00 (0.61, 1.65) | 0.98 (0.55, 1.75) | 0.97 (0.49, 1.91) | 0.82 (0.37, 1.81) |
| **Sensitivity analyses using TCR covariates^b^** | | | | |
| Q1 (least disadvantaged) | - | 1.00 | - | 1.00 |
| Q2 | - | 0.89 (0.58, 1.37) | - | 0.80 (0.46, 1.41) |
| Q3 | - | 0.86 (0.56, 1.32) | - | 0.85 (0.50, 1.46) |
| Q4 (most disadvantaged) | - | 1.02 (0.69, 1.52) | - | 0.89 (0.53, 1.50) |

Abbreviations: ADI=Area Deprivation Index; aHR=adjusted hazard ratio; cHR=crude hazard ratio; CI=confidence interval; nSES=neighborhood socioeconomic status; TCR=Texas Cancer Registry.

^a^ Adjusted for sex, age at diagnosis, race/ethnicity, cytogenetic features, National Cancer Institute risk group at diagnosis, immunophenotype, and treating institution.

^b^ Adjusted for sex, age at diagnosis, and race/ethnicity.
